# Liver CEBPβ Modulates the Kynurenine Metabolism and Mediates the Motility for Hypoxia-Induced Central Fatigue in Mice

**DOI:** 10.3389/fphys.2019.00243

**Published:** 2019-03-14

**Authors:** Fan Yang, Li Zhou, Jun Song, A WangJinMei, Yuan Yang, Zhong-wei Tang, Qing-Yuan Huang

**Affiliations:** ^1^Joint Surgery, General Hospital of Tibetan Military Command Lhasa, Lhasa, China; ^2^Department of Cold Environment Medicine, College of High Altitude Military Medicine, Third Military Medical University and Key Laboratory of High Altitude Medicine, Ministry of Education, Key Laboratory of High Altitude Medicine, PLA, Chongqing, China; ^3^Department of Pharmacy, General Hospital of Tibetan Military Command Lhasa, Lhasa, China

**Keywords:** CEBPβ, kynurenine, hypoxia, central fatigue, exercise tolerance

## Abstract

Central fatigue is defined as a failure of the central nervous system to adequately drive the muscle, manifesting limited development, and maintenance of locomotor activity. A plateau in hypoxia leads to central fatigue and followed by maximal motility recession. However, the underlying mechanism is still unclear. The present study describes a mechanism by which liver CEBPβ (CCAAT/enhancer-binding protein beta) induced by hypoxic environment alters the kynurenine (KYN) metabolism and causes the suppression of motility function recession. The activation of CEBPβ under hypoxia increases the liver expression of tryptophan dioxygenase, thereby enhancing the conversion of tryptophan into KYN; the KYN metabolite can traverse the blood-brain barrier and result in the suppression of motility function. However, the knockdown of CEBPβ by injecting pAAV-shRNA-CEBPβ via the hepatic portal vein reduces the KYN production and improves the motility function. KYN is a neurochemical that which restricts the exercise capacity after injection in the basal ganglia in mice. Reducing the plasma KYN protects the brain from hypoxia-induced changes associated with fatigue, and the knockdown liver of CEBPβ in mice renders resistance to fatigue post-acute hypoxia or tryptophan treatment. This study reveals resistance to central fatigue as a strategy for acclimatization to hypoxia mediated by transcription factor CEBPβ in the liver.

## Introduction

Fatigue can be divided into peripheral fatigue and central fatigue from different perspectives (Marquez et al., 2017; Twomey et al., 2017). Peripheral fatigue refers to the decline in muscle contraction caused by non-nerves, while the central fatigue is derived from the central nervous system and characterized by a decrease in the capacity of muscle contraction during prolonged exercise (Keyser, 2010; Marquez et al., 2017). Central fatigue is a major concern in patients with Parkinson’s (Bruno and Sethares, 2015; Lou, 2015), Alzheimer’s (da Rocha et al., 2011), cerebral tumor (Day et al., 2016), multiple sclerosis (Ayache and Chalah, 2017; Biberacher et al., 2017; Collin et al., 2017), and hypoxemia (Siebenmann and Rasmussen, 2016; Jubeau et al., 2017). The mechanism underlying central fatigue contributes toward metabolic, circulatory, and neurochemical changes in the cerebral homeostasis (Meeusen and Roelands, 2017). Plateau hypoxia can cause central fatigue and decline in motility function. For example, the ultimate treadmill running capacity reduced to 50% to that of the plain when an adult reached 4500 m high altitude (Goodall et al., 2012; Millet et al., 2012) within a short duration. Reduced oxygen partial pressure availability could facilitate central fatigue by altering the neurochemistry that plays a key role in the development of central fatigue. Thus, identifying the changes in this neurochemistry might explain the causes of hypoxic central fatigue.

Brain neurochemistry participates in central fatigue during exercise substantially (Meeusen and Roelands, 2017). Previous studies have shown that post-exercise rodents release neurotransmitters, which evoke fatigue during dopaminergic influx, and followed by interference in thermoregulation in the hypothalamus (Bridge et al., 2003; Roelands and Meeusen, 2010; Zheng et al., 2014). In addition, alleviating the levels of brain serotonergic activity due to prolonged exercise would augment lethargy and negatively influence the mood and drive, resulting in fatigue (Meeusen et al., 2006; Meeusen and Watson, 2007). However, the regulation of human central fatigue is rather complicated and unpredictable, since a majority of the studies have found that the modulation of neurotransmitters failed to alter fatigue (Struder et al., 1998; Smirmaul et al., 2017). Thus, we focus on a new neurochemical, kynurenine (KYN) that may cause central fatigue. Agudelo et al. (2014) reported that KYN could readily traverse the blood-brain barrier, leading to depression, and reduction of forced swimming time in mice. Most brain KYN originates from the peripheral tryptophan degradation pathway and expresses primarily in the liver, kidney, and immune cells (Muller and Schwarz, 2007). KYN is produced by tryptophan metabolism under tryptophan dioxygenase (TDO) or indoleamine dioxygenase (IDO) catalysis in liver and lymph, respectively (Zwilling et al., 2011; Nicoletti, 2015).

Moreover, muscle catabolism increases under hypoxic status, indicating that the increased tryptophan substrate could be degraded to KYN under TDO as a catalyst. Furthermore, an animal study found that the TDO expression in the liver increased three-fold under 8000 m acute hypoxia after 12 h in rats (Yoshino et al., 1991). In the present study, we showed that the modulation of liver KYN metabolism through CEBPβ expression mediated hypoxia-induced fatigue behavior. The transcription factor CEBPβ increases the expression of TDO in the liver; importantly, this shifts the peripheral metabolism of hypoxia-induced and exogenous tryptophan into KYN, thereby leading to central fatigue and reduction in exercise tolerance.

## Materials and Methods

### Spontaneous Activity Test

All C57BLC/6 male mice were obtained from the Laboratory Animal Center, Third Military Medical University, Chongqing, China and housed in specific pathogen-free conditions with food and water *ad libitum*. Experimental protocols were approved by the Medical Ethics Committee of the Third Military Medical University.

The spontaneous activity of mice was assessed by an open field test (ZH10954, China), performed as described previously (Alfieri et al., 2014). Briefly, the mice were placed in a clear Plexiglas (40 cm × 40 cm × 40 cm) with white floor for 10 min for acclimatization before the experiment. The locomotor activity was assessed during 5 min. The open field area was placed in the center of the room and maintained in the dark. Total distance, average speed, and total resting time were quantified.

### Wheel Running Test

The animals were tested on a 10-lane treadmill using a running wheel with inner circumference 70 cm (Iitcinc, United States). The 3 days before the experiment comprised of a habituation period for the running wheel activity, and each mouse was trained 3 times/day in a row until it fell. The parameters of the formal experiment were as follows: the total measured time was 120 min, followed by rest of 20 s after a shock, and the third rest in 5 min, which was considered fatigue; the precise time was recorded. When the animal is exhausted, it collapses to the ground and can be grabbed without resistance.

### Hypoxia

Mice of the hypoxia group were placed in a hypobaric chamber simulating an elevation of 4000 m for 24 h, followed by evaluating the behavior or sacrificed immediately.

### Gene Prediction

We found that the *TDO* gene is transcribed by CCAAT/enhancer-binding protein (CEBP) using SABioscience software analysis (Supplementary Figure S1).

### Animal Surgery and shRNA

Mice were divided into 3 groups: Normoxia+pAAV-shRNA-rScramble group, Hypoxia+pAAV-shRNA-rScramble group, and Hypoxia+pAAV-ShRNA-CEBPβ group. Animals were anesthetized with an intraperitoneal (i.p.) injection of chloral hydrate (50 mg/kg body weight). Subsequently, the abdomen was opened in the lower margin of the upper right and incision less than 1cm was made. Then, the hepatic vein was carefully isolated, 4.99 × 10^12^ μg/mL titer adeno-associated virus (AVV) packed with pAAV-ZsGreen-shRNA (Agilent, BioWit Technologies, United States) which is a specific promoter for liver was injected into the hepatic portal vein in mice. After the operation, the abdominal cavity was sutured, and the animals followed up after 1 week.

The presence of *CEBPβ* shRNA gene in recombinant viral DNA was verified by polymerase chain reaction (data not shown). The forward and reverse primer sequences were 5′-GATCCGCTGAGCGACGAGTACAAGATTTCAAGAGAATCTTGTACTCGTCGCTCAGCTTTTTTAGATCTA-3′ and 5′-AGCTTAGATCTAAAAAAGCTGAGCGACGAGTACAAGATTCTCTTGAAATCTTGTACTCGTCGCTCAGCG-3′, respectively. The scramble shRNA sequence was used as the control group: forward primer 5′-AACTTTCTCCGAACGTGTCACGTTTCAAGAGAACGTGACACGTTCGGAGAATTTTTTC-3′, reverse primer 3′-TTGAAAGAGGCTTGCACAGTGCAAAGTTCTCTTGCACTGTGCAAGCCTCTTAAAAAAGAGCT-5′.

### Real-Time Quantitative Reverse Transcription-Polymerase Chain Reaction (qRT-PCR)

Following the wheel running test in hypoxia, the mice were immediately sacrificed and the liver removed. The total RNA was extracted and reverse transcribed using SuperScript III Kit (Invitrogen, United States), and qRT-PCR was performed using a PCR instrument (MJ, Research Opticon CFD-3200, United States). PCR amplification was carried out using the speci-fic primers: *CEBPα*, forward, 5′-GCGGGAACGCAACAACATC-3′, reverse, 5′-GTCACTGGTCAACTCCAGCAC-3′; *CEBPβ*, forward, 5′-TACGAGCCCGACTGCCTG -3′, reverse, 5′-TCGGAGAGGAAGTCGTGGTG-3′; *CEBPγ*, forward, 5′-AGCGGCTTACAGCAGGTTC-3′, reverse, 5′-GGCGGTATTCGTCACTATTCC-3′; *CEBPδ*, forward, 5′-CGACTTCAGCGCCTACATTGA-3′, reverse, 5′-GAAGAGGTCGGCGAAGAGTT-3′; *CEBP𝜀*, forward, 5′-GCAGCCACTTGAGTTCTCAGG-3′, reverse, 5′-GATGTAGGCGGAGAGGTCGAT-3′. *β-actin* was used as an endogenous control, and the relative expression of the mRNA samples was calculated using the 2^-ΔΔCT^ method.

### Western Blotting

The total protein was extracted from the liver after exposure to hypoxia for 24h, and the concentrations measured using the bicinchoninic acid (BCA) protein assay. The proteins were separated by electrophoresis and transferred to PVDF membranes. Subsequently, the Western blotting was carried out by standard protocol. The membranes were probed with primary antibodies: anti-CEBPβ (1:500, Abcam, United States), anti-TDO-1 (1:300, Abcam), anti-TDO-2 (1:500, Abcam), anti-KAT-1 (1:200, Abcam), and anti-β-actin (1:1000 dilution, Santa Cruz Biotechnology, Santa Cruz, CA, United States). The immunoreactive bands were visualized using the enhanced chemiluminescence (ECL) kit according to the manufacturer’s instructions (Amersham Biosciences, Arlington Heights, IL, United States). The signal intensities of the target proteins were analyzed by the bioimaging system (Model 4000, Versa Doc, Bio-Rad, United States) and the densitometric values analyzed by Image J software. The housekeeping protein β-actin served as an internal control.

### Immunohistochemistry

Liver tissue samples were fixed in 4% formaldehyde, dehydrated in 30% sucrose solution, and sliced into 20-μm-thick sections using Leica Microsystems Nussloch GmbH (D-69226, Germany). After blocking with 10% normal goat serum, the sections were permeabilized using 0.1% Triton-X 100 and incubated with primary antibody anti-CEBPβ (1:100, Abcam). After washing, the samples were probed with the appropriate secondary antibody (Jackson Immunoresearch, West Grove, PA, United States). Micrographs were selected, captured using a laser confocal microscope and analyzed using MagnaFire SP 2.1B software (Olympus, Melville, NY, United States).

### High-Performance Liquid Chromatography (HPLC)

Kynurenine and tryptophan levels in serum were analyzed by HPLC, as described previously (Engin et al., 2015). Briefly, loading of the serum on the column at a flow rate 1 ml/min, at 22°C. The mobile phase consisting of 0.1M ammonia acetate (pH 4.65) was filtered prior to usage and pumped isocratically at a flow rate of 0.8 ml/min. Tryptophan was measured by fluorescence detection at an excitation wavelength of 254 nm and an emission wavelength of 404 nm, while KYN was measured using a multi-wavelength detection at 365 nm. The final results were calculated according to the standard curve.

### Intracranial Catheterization and KYN Injection

After the mice were anesthetized with an i.p. injection of chloral hydrate (100 mg/kg body weight) and placed in a stereotaxic apparatus, a guide cannula (AG-8; Eicom, Tokyo, Japan) was implanted into the basal ganglia (coordinates: 0.2 mm anterior, 5.5 mm ventral, and 3.5 mm lateral to the midline), and fixed to the skull with dental cement and small screws, according to the coordinates provided by Klippel’s atlas. During the 1-week postoperative recovery period, the mice were acclimated to handling and experimental cage used for 1, 3, and 9 mg KYN administration, followed by behavioral tests in half an hour. A total of 45 mice were cannulated, of which, 5 were excluded from the study because of cannula misplacement.

### Statistical Analysis

Data were analyzed by SPSS 13.0 software (SPSS Inc., Chicago, IL, United States). The data are presented as a mean ± standard deviation. *Post hoc* test was used for the comparison of two groups. Statistical significance among different groups was determined using one-way analysis of variance. *P* < 0.05 was considered as statistically significant.

## Results

### Elevated Liver CEBPβ Expression in Acute Hypoxia Impacts Fatigue Responses

**FIGURE 1:**
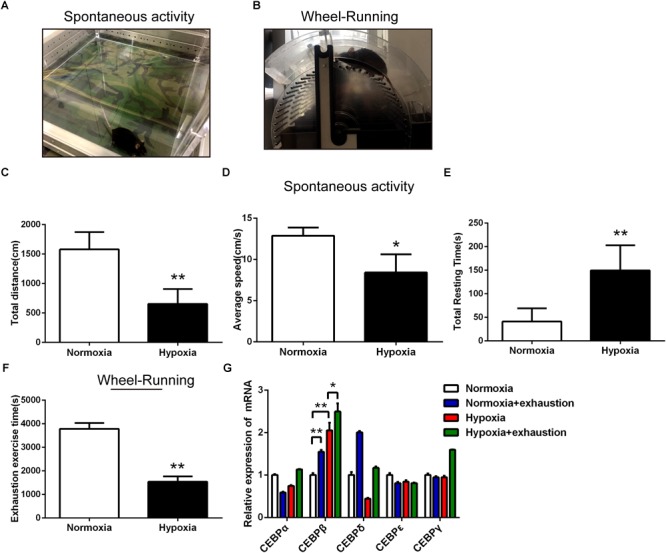
The motility decreased and the mRNA expression of CEBPβ increased under 4000 m hypoxia. Spontaneous activity test **(A)** and wheel running test **(B)** were used to evaluate the motility in mice. Total distance **(C)**, average speed **(D)**, and total resting time **(E)** were measured within 5 min in mice in spontaneous activity test in the dark under hypoxia (*n* = 10 for each group, ^∗^*P* < 0.05, ^∗∗^*P* < 0.01). The exhaustion exercise time **(F)** was calculated by the wheel running test in illumination under 4000 m hypoxia (*n* = 10 for each group, ^∗∗^*P* < 0.01). **(G)** The mRNA expression of CEBP (five different subtypes) in the liver after exhaustion in the wheel running test under 4000 m hypoxia (*n* = 6 for each group, ^∗^*P* < 0.05, ^∗∗^*P* < 0.01).

First, we determined the locomotor activity and forced exhaustive exercise through spontaneous activity test (Figure 1A) and wheel running test (Figure 1B) in mice under 4000 m hypoxia, respectively. Animals suffered from 24 h hypoxia before behavioral testing. Our data showed that the total distance (Figure 1C) and average speed (Figure 1D) were shortened significantly, while the total resting time (Figure 1E) was prolonged in the spontaneous activity test. Moreover, the exhaustion exercise time was also reduced as assessed by the wheel running test (Figure 1F). As reported previously (Fan and Kayser, 2016; Marillier et al., 2017), both the locomotor activity and the maximal exercise tolerance of the animals decreased under hypoxic environment. We also examined the mRNA expression of CEBP family (α, β, γ, δ, and 𝜀) in the liver in different states of motion under hypoxia. Notably, the CEBPβ mRNA increased in Normoxia+exhaustion and Hypoxia groups as compared to the Normoxia group mice. CEBPβ mRNA in Hypoxia+exhaustion group increased as compared to the Hypoxia group (Figure 1G). These results suggested that both hypoxia and exhaustive exercise can significantly increase the mRNA expression of CEBPβ in the liver.

### CEBP Induces the Expression of TDO in the Liver Under Hypoxia

**FIGURE 2:**
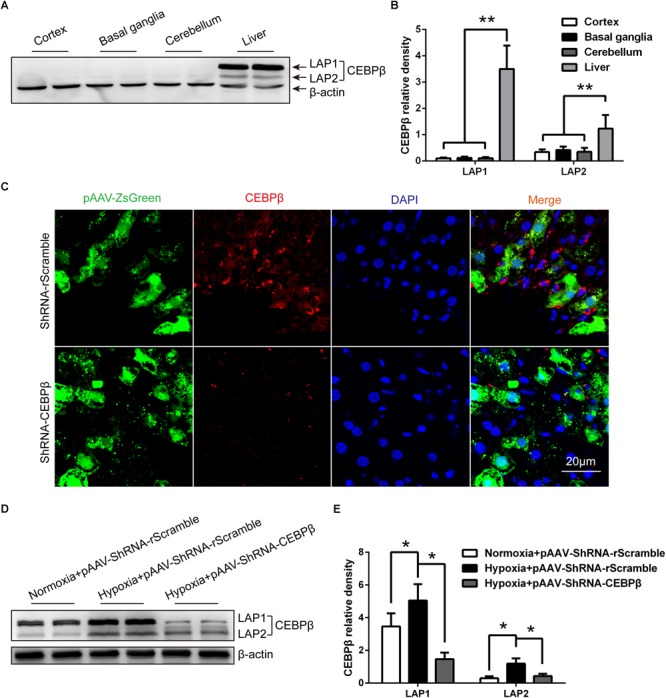
Hypoxia increased the protein expression of CCAAT/enhancer-binding protein β (CEBPβ), which is knocked down by shRNA in the liver under hypoxia. **(A)** CEBPβ protein expression in the cortex, basal ganglia, cerebellum, and liver in mice in normoxia. Liver-enriched activator protein (LAP), both LAP1 and LAP2 are two isoforms of CEBPβ. **(B)** The relative protein expression was calculated (*n* = 6 for each group, ^∗∗^*P* < 0.01). **(C)** Adeno-associated virus (pAAV-ZsGreen) and CEBPβ (red) within the liver tissue of mice after pAAV-ZsGreen-shRNA injection in hepatic portal vein for 1 week in normoxia. Nuclei were counterstained with DAPI (blue). Scale bar, 20 μm. **(D)** CEBPβ protein expression in the liver was detected by Western blot at 24 h under 4000 m hypoxia. **(E)** The relative protein expression was calculated (*n* = 6 for each group, ^∗^*P* < 0.05).

We examined the protein expression of CEBPβ in the liver and brain. As reported previously (Ramji and Foka, 2002), CEBPβ protein is expressed in the liver, but rarely in the central nervous system of the rodents, including cortex, basal ganglia, and cerebellum (Figure 2A,B). CEBPβ is a transcription factor that regulates the cellular growth, differentiation, metabolism, and inflammation (Ramji and Foka, 2002). Thus, to substantiate the function of CEBPβ in the liver, a specific CEBPβ pAAV-ZsGreen-shRNA (Supplementary Figure S2) was injected in the hepatic portal vein 1 week before the experiment; a majority of the cells were infected with the adeno-associated virus (AVV) in the liver, and CEBPβ was markedly knocked down as evaluated by immunofluorescence (Figure 2C) and Western blot (Figure 2D). In addition, not only the mRNA level but also the protein level of CEBPβ increased in the liver after 24 h under 4000 m hypoxia (Figure 2D,E).

**FIGURE 3:**
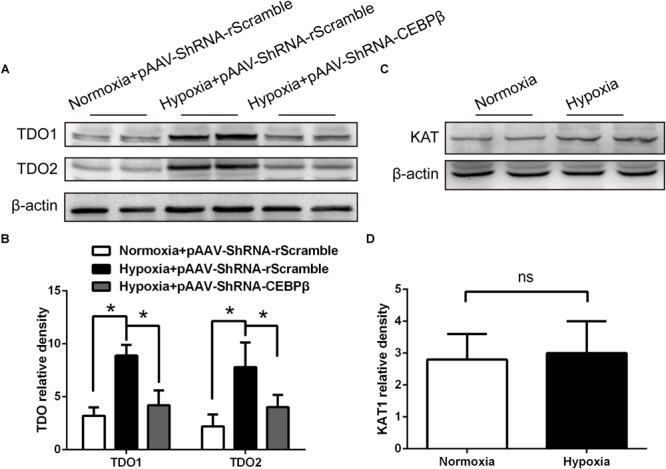
Knockdown of CEBPβ reduces the expression of tryptophan dioxygenase (TDO) in the liver under hypoxia. **(A)** TDO1 and TDO2 protein expression in the liver were detected by Western blot when CEBPβ was knocked down at 24 h under 4000 m hypoxia. **(B)** The relative protein expression of TDO was calculated (*n* = 6 for each group, ^∗^*P* < 0.05). **(C)** Kynurenine aminotransferase (KAT) protein expression in the liver was detected by Western blot at 24 h under hypoxia. **(D)** Relative protein expression of KAT was calculated (*n* = 6 for each group, ns: no statistical significance).

We hypothesized that the hypoxia-induced central fatigue was associated with the activation of CEBPβ, which regulates TDO transcription, thereby enhancing the conversion of tryptophan into KYN; KYN is a metabolite that can cross the blood-brain barrier and trigger central fatigue (Zwilling et al., 2011; Nicoletti, 2015). In order to investigate the relationship between CEBPβ and TDO, the protein expression of TDO was determined. We found that both TDO1 and TDO2 protein expression significantly increased under 4000 m hypoxia; this phenomenon was reversed when CEBPβ was knocked down (Figure 3A,B). We also detected that kynurenine aminotransferase (KAT) catalyzed the metabolism of KYN to kynurenic acid (KYNA) and remained unaltered under hypoxia (Figure 3C,D). These results suggested that CEBPβ participates in TDO protein transcription.

### Liver CEBPβ Enhances the Peripheral Metabolism of Tryptophan to KYN During Hypoxia

**FIGURE 4:**
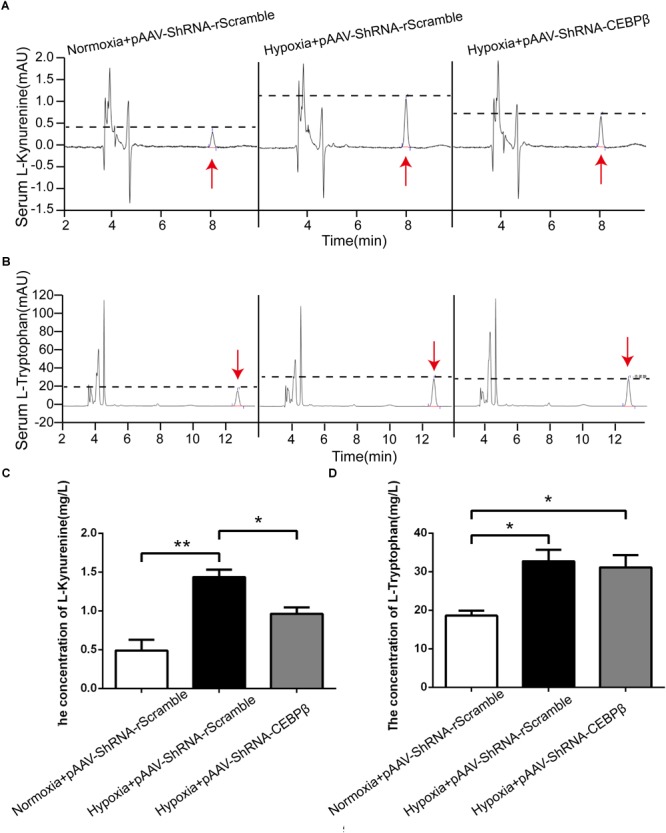
Knockdown of CEBPβ reduces the production of kynurenine (KYN) under hypoxia. KYN **(A)** and TRP **(B)** levels in serum were detected by High Performance Liquid Chromatography (HPLC) when CEBPβ was knocked down. The relative levels of KYN **(C)** and TRY **(D)** were calculated according to the standard substance (*n* = 6 for each group, ^∗^*P* < 0.05, ^∗∗^*P* < 0.01).

CEBPβ knock-down mice model was utilized for the further investigation of the levels of KYN and tryptophan in the periphery. As expected, HPLC data revealed that KYN increased considerably in the serum at 24 h under hypoxia, while it was decreased when CEBPβ was knocked down (Figure 4A,C). Tryptophan, a precursor of KYN production, derived from the endogenous amino acids in tissue protein decomposition, constituted approximately 2/3 of the total Tryptophan (Yamashita and Yamamoto, 2014; Malhotra et al., 2017). We observed that tryptophan added after hypoxia in the liver of mice, but it was unacted on CEBPβ knockdown (Figure 4B,D). These results implied that KYN metabolic pathway is regulated by CEBPβ through TDO in the liver.

**FIGURE 5:**
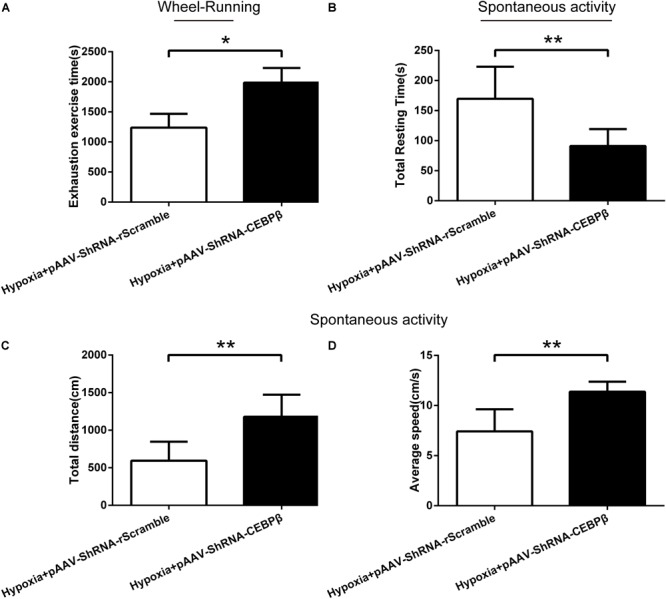
Knockdown of CEBPβ improves the motility in mice under hypoxia. **(A)** Exhaustion exercise time was calculated by the wheel running test in illumination under 4000 m hypoxia (*n* = 10 for each group, ^∗^*P* < 0.05). The total resting time **(B)**, total distance **(C)**, and average speed **(D)** was measured within 5min in spontaneous activity test in the dark under hypoxia (*n* = 10 for each group, ^∗∗^*P* < 0.01).

To confirm the effect of liver CEBPβ knockdown on the motility in mice, behavioral tests were employed. Our data showed that the exhaustion exercise time was prolonged significantly (Figure 5A) in the wheel running test when CEBPβ was knocked down. Moreover, the total resting time (Figure 5B) was shortened, while the total distance (Figure 5C) and average speed (Figure 5D) were prolonged significantly as assessed by the spontaneous activity test. These results suggested that the inhibition of CEBPβ could enhance the exercise tolerance and locomotor activity in the liver of mice under hypoxia.

### Central KYN Administration Reduces the Motility Capacity

**FIGURE 6:**
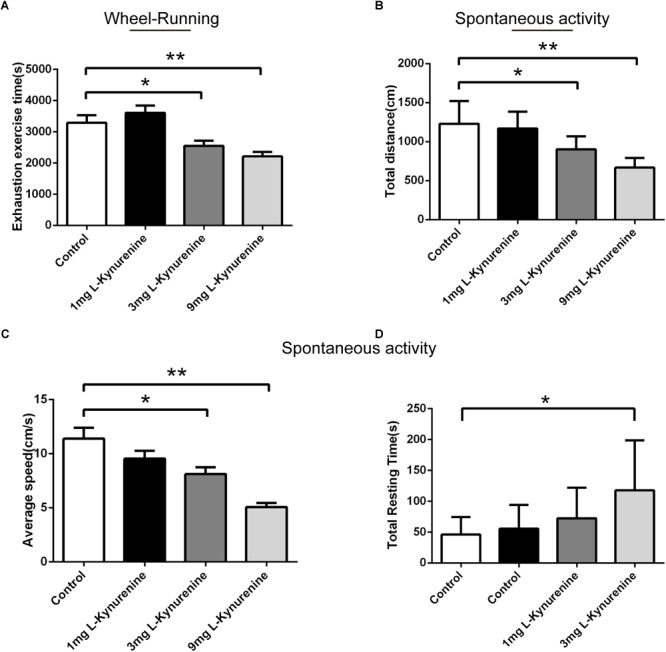
Central kynurenine administration reduced the motility capacity. **(A)** Exhaustion exercise time was calculated in the wheel running test in illumination after administration of 1, 3, and 9 mg KYN (*n* = 10 for each group, ^∗^*P* < 0.05, ^∗∗^*P* < 0.01). Total resting time **(B)**, total distance **(C)**, and average speed **(D)** were measured within 5mins in spontaneous activity test in the dark after administration of 1, 3, and 9 mg KYN (*n* = 10 for each group, ^∗^*P* < 0.05, ^∗∗^*P* < 0.01).

We confirmed that the upregulation of CEBPβ increases the expression of TDO and the production of KYN in the liver under hypoxia. Previous reports demonstrated that the central accumulation of KYN was correlated to the postoperative- (Chen et al., 2015), depression- (Agudelo et al., 2014; Malhotra et al., 2017; Serafini et al., 2017), and tumor (Cathomas et al., 2015; Day et al., 2016)-related central fatigue. In order to clarify the role of KYN in central fatigue, KYN was injected into the basal ganglia, which is the core of motor control connecting the thalamus and the frontal cortex (Leisman et al., 2016). We observed that both 3 and 9 mg KYN could significantly decrease the exhaustion exercise time (Figure 6A). In addition, although the total distance (Figure 6B) and average speed (Figure 6C) were shortened, the total resting time (Figure 6D) was prolonged. These results demonstrated that KYN could affect the central nervous system, resulting in a decreased motor performance.

### Peripheral Tryptophan Administration Decreases the Motility Capacity Depending on CEBPβ

**FIGURE 7:**
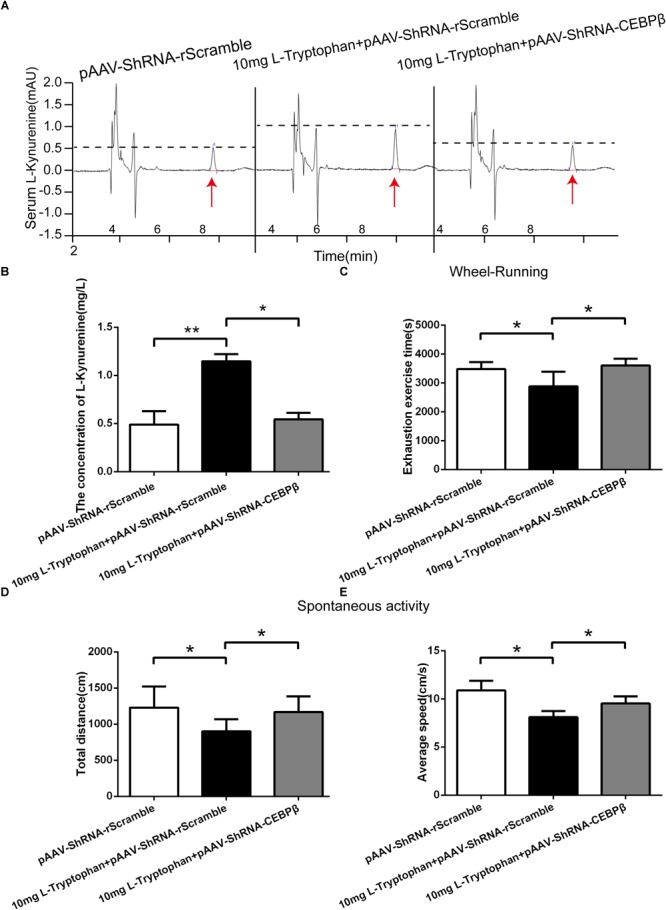
CEBPβ manipulates the metabolism of TRP to KYN related to the motility in mice. **(A)** KYN levels in serum were detected at 24 h after intraperitoneal injection 10 mg TRP in CEBPβ knockdown mice. **(B)** Relative levels of KYN were calculated based on the standards (*n* = 6 for each group, ^∗^*P* < 0.05, ^∗∗^*P* < 0.01). **(C)** Exhaustion exercise time was calculated by the wheel running test in illumination at 24 h post-intraperitoneal injection of 10 mg TRP in CEBPβ knockdown mice (*n* = 6 for each group, ^∗^*P* < 0.05). The total distance **(D)** and average speed **(E)** were measured within 5 min in spontaneous activity test in the dark at 24 h after intraperitoneal injection of 10 mg TRP in CEBPβ knockdown mice (*n* = 6 for each group, ^∗^*P* < 0.05).

Our data suggested that hypoxia-induced increase in serum KYN levels have central effects associated with fatigue. Moreover, the KYN plasma levels directly correlated with hepatic CEBPβ and TDO. Furthermore, we determined the effects of CEBPβ on TRP degradation and KYN production. The i.p. injection of 10mg tryptophan remarkably increased the peripheral KYN levels after 24 h in normoxia (Figure 7A,B). In addition, the exhaustion exercise time (Figure 7C), total distance (Figure 7D), and average speed (Figure 7E) were shortened after tryptophan administration. However, the effects of tryptophan were partially attenuated when CEBPβ was knocked down (Figure 7).

## Discussion

As a consequence of exposure to high altitude, the decline in the exercise performance is one of the most enduring effects of hypoxia on the human body, which indicates early fatigue. CCAAT/enhancer-binding protein β (CEBPβ), a stress-responsive transcription factor activated by hypoxia, promotes inflammatory response (Villar et al., 2012), apoptosis, and lymphangiogenesis (Min et al., 2011). CEBPβ is primarily up-regulated in athletic exhaustion as compared to sedentary state irrespective of the horses(Cappelli et al., 2013) or humans (Blackwell et al., 2015). In the present study, the expression of CEBPβ in the liver, rather than the central nervous system, was determined, which resulted in the following conclusions: Firstly, increased CEBPβ expression promotes the production of TDO and KYN. Secondly, CEBPβ knockdown improves the motility capacity in mice under 4000 m hypoxia. Thirdly, striatal injections of KYN can mimic the hypoxia-induced the reduction of motor capacity.

Fatigue effect is subjective, including peripheral and central fatigue. Central fatigue is a muscular asthenia regulated by motor neurons from the central nervous system, which may be predicted by classical models of physiological maxima. The presence of central fatigue under the hypoxic condition is identified gradually recognized although sequestered (Fan and Kayser, 2016; Jubeau et al., 2017). In the case of hypoxia, the sensitive areas of the brain can perceive this change and restrain the locomotor activities of the related target (Fan and Kayser, 2016). For example, the excitability of supraspinal and corticospinal neurons was inhibited after high-altitude exposure in rats (Marillier et al., 2017). Similarly, the restriction of exercise performance is due to the failure of the drive from the motor cortex in the case of acute severe hypoxia, potentially as a consequence of diminished O_2_ availability in the brain (Goodall et al., 2012; Millet et al., 2012).

Protein catabolism is higher than synthetic metabolism under hypoxia, leading to an increased aminogram in the blood, which can give rise to central fatigue. Recent studies suggested that exercise-induced changes in the concentrations of serotonin (5-HT), dopamine (DA), and noradrenaline (NA) contribute to the onset of fatigue during prolonged exercise. 5-HT has been shown to increase during acute exercise in running rats and remain high at the point of fatigue. DA release is also elevated during exercise but appears to decline at the point of exhaustion, in which may be crucial for the fatigue process. Notably, KYN, a tryptophan metabolite, can penetrate the blood brain barrier and arouse central effects, such as depression (Agudelo et al., 2014), reduction of locomotor activity, and myasthenia (Zwilling et al., 2011). On the contrary, transgenic murine models with low-level KYN show fatigue-resistant phenotype (Choi et al., 2008). Furthermore, we confirmed that KYN produced central fatigue and decreased motor capacity and that KYN was significantly increased after hypoxia. These results suggested that KYN might be involved in hypoxia-induced central fatigue.

Kynurenine is produced in the liver and reaches the brain via blood circulation (Agudelo et al., 2014). It is readily transported into the brain tissue via the transporter-dependent or non-transporter-dependent manner and accumulates in the intercellular space and synaptic junctions of the neurocytes (Agudelo et al., 2014; Malhotra et al., 2017). KYN significantly inhibits the excitability of central motor neurons and decreases the contraction of the centrally driven skeletal muscle, resulting in short exhaustion exercise time. KYN can be further degraded to KYNA catalyzed by KATs. As the movement stopped, the production of KYN in the liver is decreased, and the central inhibitory effect of KYN is gradually relieved.

**FIGURE 8:**
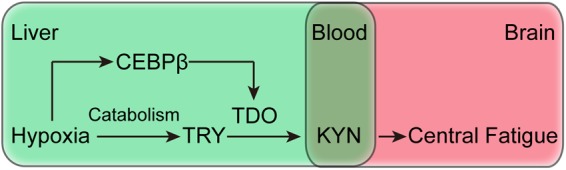
Schematic of the mechanism of CEBPβ and the effects of central fatigue, which regulates the peripheral metabolism of TRP to KYN by TDO.

Investigating the causes of increased KYN production is valuable in understanding the central fatigue in hypoxia. Moreover, KYN is produced by the catalysis of tryptophan in TDO or IDO. TDO is mainly expressed in the liver, whereas IDO is expressed in the lymph nodes(Ito et al., 2003). On the other hand, brain tissues do not express TDO and IDO, and hence, the central nervous system produces KYN rarely (Ito et al., 2003). Although tryptophan can also produce KYN under the catalysis of IDO, there is no evidence that the expression and activity of IDO protein increases under hypoxic conditions. Therefore, the increase of KYN in hypoxic conditions is primarily caused by hepatic TDO catalysis. Herein, we report for the first time that the effects of liver CEBPβ on KYN production under hypoxia. Both hypoxia and exhaustive exercise can independently or synergistically induce the increase of CEBPβ expression in the liver. Conversely, the inhibition of CEBPβ expression can increase the locomotor activity and exercise capacity. Interestingly, after knocking down liver CEBPβ, accompanied by a decrease in TDO, the production of KYN is also decreased (Figure 8). These results suggest that the generation of KYN is related to CEBPβ. In the case of hypoxia, CEBPβ increases the transcription of TDO protein, thereby promoting the decomposition of tryptophan into KYN.

Many of the important metabolites of the KYN metabolism pathway in central fatigue will be authenticate in experimental animals by an intrastriatal injection of the TRP in CEBPβ knockdown mice in the future. KYN metabolism is dependent on a series of enzymatic reactions. In short, KYN can be catalyzed to generate KYNA by Kynureni aminotransferase, or can be metabolized to generate 3-hydroxykynurenine (3-HK) by Kynurenine 3-monooxygenase (KMO), and then further degraded to Quinolinic acid (QUIN) and end up with NAD+(Zwilling et al., 2011). A randomized, double-blind placebo controlled trial reported that oral CoQ10 plus NADH supplementation could significant improvement of fatigue showing a reduction in fatigue impact scale total score on Chronic fatigue syndrome patients (Castro-Marrero et al., 2015). Therefore, investigating the changes in KYN metabolic downstream pathways, as well as the further induction of NAD by KYN that transports the liver to the central nervous system, may contribute to the improvement of central fatigue under hypoxia conditions.

About a decade after the original central fatigue hypothesis, animal studies appear to be able to alter the fatigue in normal environmental conditions using pharmacological manipulations of brain neurotransmission (Meeusen et al., 2006). However, some conclusions do not apply to humans, indicating the participation of rather sophisticated mechanisms that are to be elucidated (Meeusen et al., 2006; Meeusen and Roelands, 2017). Moreover, intracranial temperature, as a vital factor in central fatigue, is often overlooked. For example, the injection of dual DA and NA reuptake inhibitor induce a significant increase in brain and core temperature with a decrease in heat loss responses (Hasegawa et al., 2005, 2008).

Hypoxia can cause significant fatigue and decrease the exercise capacity, which is a complex process involving multiple organs and mechanisms (Fan and Kayser, 2016; Siebenmann and Rasmussen, 2016; Marillier et al., 2017). In order to confirm the effects of peripheral KYN on central fatigue, we administered pre-hypoxia for 24 h before animal movements. Under hypoxia, the KYN produced by the liver acts on the central motor neurons, which essentially belongs to the humoral feedback between the peripheral organs and central nervous system. KYN can combine with specific receptors on motor neurons, such as N-methyl-D-aspartate receptor (Chen et al., 2015; Kumar, 2015); however, the central fatigue effect of KYN needs to be further elucidated.

In summary, we show that the modulation of liver KYN metabolism through CEBPβ expression mediates the hypoxia-induced fatigue behavior. The transcription factor CEBPβ increases the expression of TDO in the liver. Importantly, this shifts the peripheral hypoxia-induced metabolism and exogenous tryptophan into kynurenine, thereby leading to central fatigue and the reduction of exercise tolerance.

## Author Contributions

FY, LZ, JS, AW, YY, and Z-wT carried out the experiments. FY, LZ, and JS analyzed the data. FY and LZ designed the experiments. FY and Q-YH wrote the manuscript.

## Conflict of Interest Statement

The authors declare that the research was conducted in the absence of any commercial or financial relationships that could be construed as a potential conflict of interest.

## Supplementary Material

FIGURE S1 Prediction of transcription start site of TDO with CEBPβ by SABioscience software.

FIGURE S2 Construction of pAAV-ZsGreen-shRNA cloning vector for *Streptococcus thermophiles*.
