# Supplementary figures and images for: Liver CEBPβ Modulates the Kynurenine Metabolism and Mediates the Motility for Hypoxia-Induced Central Fatigue in Mice
Source: Front Physiol. 2019 Mar 14;10:243. doi: 10.3389/fphys.2019.00243 (PMC6428026; doi:10.3389/fphys.2019.00243)

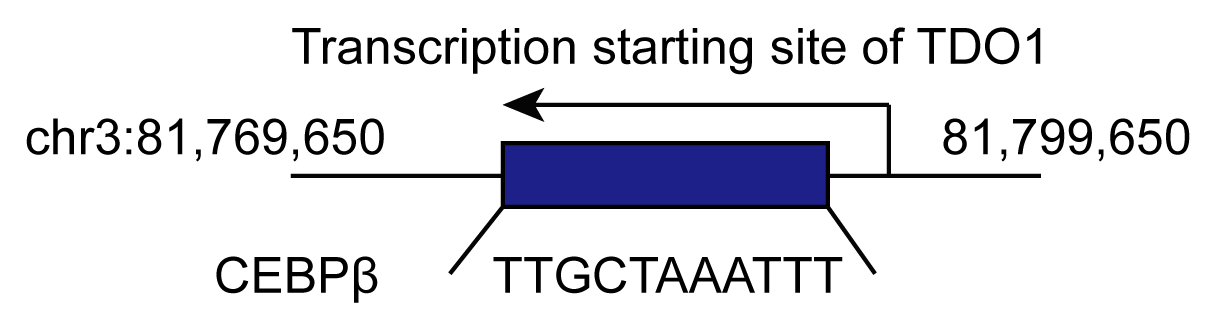

Supplement: FIGURE S1 — Prediction of transcription start site of TDO with CEBPβ by SABioscience software. [file Image_1.TIF]

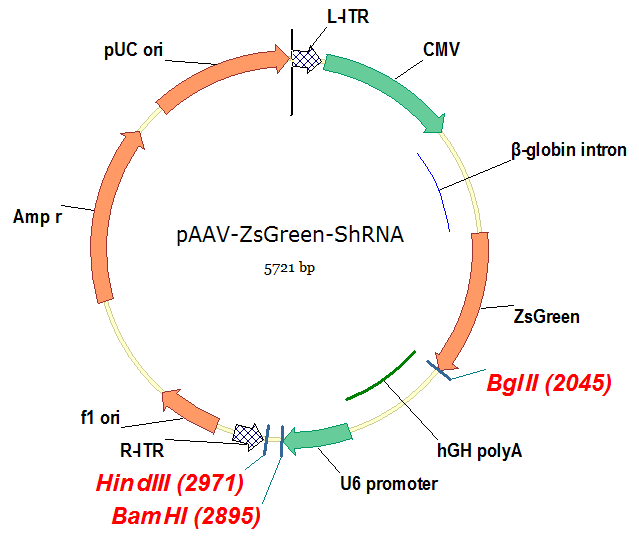

Supplement: FIGURE S2 — Construction of pAAV-ZsGreen-shRNA cloning vector for Streptococcus thermophiles. [file Image_2.TIF]
